# An Integrated Perspective on Virulence-Associated Genes (VAGs), Antimicrobial Resistance (AMR), and Phylogenetic Clusters of Pathogenic and Non-pathogenic Avian *Escherichia coli*

**DOI:** 10.3389/fvets.2021.758124

**Published:** 2021-11-24

**Authors:** Seyede Elham Rezatofighi, Arash Najafifar, Mahdi Askari Badouei, Seyed Mostafa Peighambari, Mohammad Soltani

**Affiliations:** ^1^Faculty of Science, Department of Biology, Shahid Chamran University of Ahvaz, Ahvaz, Iran; ^2^Private Veterinary Practitioner, Independent Researcher, Tehran, Iran; ^3^Faculty of Veterinary Medicine, Department of Pathobiology, Ferdowsi University of Mashhad, Mashhad, Iran; ^4^Faculty of Veterinary Medicine, Department of Avian Diseases, University of Tehran, Tehran, Iran

**Keywords:** APEC, AFEC, virulence, AMR, typing, phylogroup, Iran

## Abstract

Avian pathogenic *Escherichia coli* (APEC) is an important bacterial pathogen that causes avian colibacillosis and leads to huge economic losses in the poultry industry. Different virulence traits contribute to pathogenesis of APEC infections, and antimicrobial resistance (AMR) has also been an overwhelming issue in poultry worldwide. In the present study, we aimed to investigate and compare the presence of virulence-associated genes (VAGs), AMR, and phylogenetic group's distribution among APEC and avian fecal *E. coli* (AFEC) strains. *E. coli* from birds with colisepticemia and yolk sac infection (YSI) (APEC) plus *E. coli* strains from the feces of healthy birds (AFEC) were compared by the aforementioned traits. In addition, the clonal relatedness was compared using Enterobacterial repetitive intergenic consensus PCR (ERIC-PCR). Although all strains were susceptible to fosfomycin, ceftriaxone, and cefixime, almost all strains (98%) were multi-drug resistant (MDR). All strains (except two) harbored at least three or more VAGs, and the virulence scores tended to be higher in pathogenic strains especially in the colisepticemic group. All phylogenetic groups were found in isolates from YSI, colisepticemia, and the feces of healthy birds; however, the frequency of phylogroups varied according to the source of the isolate. B1 and C phylogroups were statistically more likely to be found among APEC from YSI and colisepticemic *E. coli* groups, respectively, while phylogroup A was the most frequently occurring phylogroup among AFEC strains. Our findings also revealed that AMR and VAGs are not essentially co-evolved traits as in some instances AMR strains were more prevalent among AFEC. This reflects the divergent evolutionary pathways of resistance acquisition in pathogenic or non-pathogenic avian *E. coli* strains. Importantly, strains related to phylogenetic group C showed higher virulence score and AMR that requires further attention. To some extent, ERIC-PCR was able to group strains by isolation source, phylogroup, or virulence genes. Further integrated studies along with assessment of more detailed genotypic and phenotypic features could potentially lead to better understanding of virulence, resistance, and evolution of ExPEC.

## Introduction

Avian pathogenic *Escherichia coli* (APEC), the etiologic agent of avian colibacillosis, is one of the most important bacterial diseases of domestic poultry causing huge economic losses worldwide (1). The disease is characterized by various conditions including septicemia, swollen head syndrome, yolk sac infection (YSI), cellulitis, as well as inflammation of different organs such as pericarditis, airsacculitis, and perihepatitis (2). APEC infections lead to increased morbidity, mortality, and carcass condemnation (3).

Virulence traits are important in the pathogenesis and epidemiology of APEC infections (4). Generally, the *E. coli* strains isolated from lesions of colibacillosis and that harbor virulence-associated genes (VAGs), such as those encoding adhesins, toxins, invasins, iron-scavenging systems, and serum survival, are classified as APEC (5, 6).

Combinations of several VAGs are needed to confer pathogenicity upon APEC and usually no single VAG alone is attributed to disease in chickens (5). According to the literature, certain serotypes appear to be associated with virulence among APEC as most O78 and O2, followed by O18 and O1 are responsible for more than half of the cases of colibacillosis worldwide (5).

From a one health perspective, APEC also appears to have a role in human disease. APEC is a subgroup of extraintestinal pathogenic *E. coli* (ExPEC), which is distinct from commensal and diarrheagenic *E. coli* groups. Different studies reported that APEC and ExPEC strains share common characteristics in terms of serotypes, phylogenetic groups, virulence factors, and ability to cause disease in various models of human and animal disease (7–9) and that *E. coli* with ExPEC characteristics occur on retail poultry meat targeted for human consumption (10). Therefore, APEC strains may represent a zoonotic threat either by causing disease in human hosts or *via* horizontal gene transfer of plasmid-linked VAGs to human commensal strains (11, 12). Importantly, many of the VAGs known to contribute to APEC virulence are linked to pathogenicity islands (PAIs) found on large transmissible plasmids (13, 14). Such plasmids may enable commensal strains to cause disease in models of human and animal disease (15, 16).

Another reason for the importance of APEC strains is the increasing antimicrobial resistance (AMR) among them. Increasing AMR, in both animal and human bacteria, has led to growing concerns worldwide (17). Multi-drug resistant (MDR) bacteria are frequently reported in poultry (17, 18). Antibiotics are administered as growth promoters and for the treatment, control, and prevention of bacterial infectious diseases in the poultry industry (19). Altogether, these factors have resulted in the higher AMR index in the poultry industry (3). These resistant strains could be the source of AMR for humans *via* chicken products or direct contact with infected birds (20).

There are different viewpoints on the relationship between VAGs and AMR. Some researchers believe that VAGs of *E. coli* strains are more associated with AMR (21, 22), whereas other researchers have believed that VAGs may even be weakly or negatively linked to AMR (23, 24).

In the present study, we aimed to reach a broader perspective on the presence of VAGs and AMR by comparisons of characteristics among APEC and avian fecal *E. coli* (AFEC) from apparently healthy birds.

## Materials and Methods

### Sample Collection and Isolation of *E. coli*

In this study, a total of 100 isolates that were previously recovered from 23 broiler farms in the Semnan province of Iran were investigated. The samples of YSI, septicemia, and healthy chickens were obtained from 8, 10, and 5 farms within the region, respectively. Since there were no colibacillosis-free farms, the healthy chicken samples were obtained from farms with the lowest mortality rate due to bacterial or mixed infections.

In brief, the specimens were obtained from the ceca fluid of apparently healthy chickens (AFEC) and chickens with typical clinical and pathological signs of colisepticemia and YSI. Septicemic cases with perihepatitis or pericarditis were sampled from liver and heart blood of broiler carcasses, respectively, while chickens with YSI were sampled from the yolk sac at necropsy examinations. The samples were streaked on MacConkey agar (Merck, Germany) and incubated at 37°C for 24 h. For identification of *E. coli* isolates, the suspected lactose-fermenting colonies were subjected to conventional biochemical tests including sugar fermentation (using TSI medium), citrate, methyl red, Voges-Proskauer (MR-VP), and production of urease, lysine decarboxylase, and indole (25). Finally, one confirmed *E. coli* isolate from each bird was selected and considered as the representative strain of that case for the further analysis.

### Antimicrobial Susceptibility Pattern

Antimicrobial susceptibility patterns of isolates were determined by the disk diffusion method according to Clinical Laboratory Standards Institute against 15 antimicrobials of different families (26, 27). For florfenicol, the breakpoints of chloramphenicol were considered since there was no available breakpoint in the CLSI guideline. The antimicrobials were chosen according to widely used drugs in animals in Iran. Also, some antimicrobials of human medicine that were illegally used by some farmers were included. The antimicrobial agents used in this study included enrofloxacin (5 μg), flumequine (30 μg), cefixime (5 μg), neomycin (30 μg), streptomycin (10 μg), gentamicin (10 μg), lincospectin (200/15 μg), chloramphenicol (30 μg), florfenicol (30 μg), furazolidone (100 μg), tetracycline (30 μg), sulfamethoxazole+trimethoprim (125/23.7 μg), ceftriaxone (30 μg), Fosbac™ (fosfomycin; 200 μg), and cefazolin (30 μg). All antimicrobial discs were purchased from Padtan Teb (Iran).

### Molecular O—Serogrouping

All *E. coli* isolates were investigated for O antigens prevalent in APEC strains including O1, O2, O18, and O78 by a previously developed multiplex-PCR assay (28).

### Phylogenetic Analysis and Virulence-Associated Genes

Bacterial DNA of isolates was extracted using the boiling method (29). Phylogenetic classification was performed according to revised PCR-based method developed by Clermont et al. (30). Old phylogenetic grouping classified isolates into four groups (A, B1, B2, and D) based on the presence or absence of *yjaA* and *chuA* genes and the TspE4.C2 DNA fragment. In revised phylogenetic analysis, the *arpA* gene is also used to assign isolates to one of eight groups: A, B1, B2, C, D, E, F, or clade I (30).

*E. coli* strains were investigated for the presence of the most significant VAGs associated with the APEC pathotype using the primers and PCR conditions previously described (31–36). The selected genes were related to iron acquisition (*iutA* and *iroN*), toxins/colicins (*hlyA, colV*, and *astA*), serum resistance (*iss* and *traT*), bacterial adhesins (*tsh, csg, papC*, and *papG*), and invasion factors (*ibeA* and *ompT*). The total number of detected virulence genes in each strain was considered as the isolate virulence score (VS). The confirmed positive controls were provided from Ferdowsi University of Mashhad (FUM) and University of Tehran microbial archives.

### Enterobacterial Repetitive Intergenic Consensus PCR (ERIC-PCR)

Fingerprinting of isolates was performed using ERIC-PCR as described previously (37). To analyze the data, the band patterns in gel electrophoresis were presumed as a binary matrix based on the presence or absence of bands with the score of 1 or 0, respectively. Dendrogram was constructed using the SIMQUAL program in NTSYS-pc, version 2.02e. The level of similarity between isolates was calculated using Jaccard's similarity coefficients and unweighted-pair group method with arithmetic averages (UPGMA).

### Statistical Analysis

Comparisons of the frequency between different groups were determined using chi-square and Fisher's exact tests with SPSS 26.0 software. Findings with *p* < 0.05 were considered significant. Comparison of the scores was determined by Mann-Whitney *U*-test using the statistical program GraphPad Prism, version 8, software (GraphPad Software, Inc., San Diego, CA). Correlation between VGs and phenotypic resistance to antibiotics was measured using Spearman's correlation coefficient matrix. Results visualized using a heatmap drawn by GraphPad Prism 8. Only correlations with a *p* < 0.05 were considered significant. For finding the most significant differences in each group, the highest adjusted residual values exceeding ±2 were indicated by (^*^) where appropriate in tables to highlight the cells contributing to the highest adjusted differences.

To classify the strains according to VAGs and AMR patterns, a dendrogram was constructed using the SIMQUAL. The presence or absence of VAGs and resistance against antimicrobial agents was considered as the score of 1 or 0, respectively. A dendrogram was also drawn according to VAG profile of the strains.

To show the distribution of the strains according to the isolation source and phylogenetic group, a circle was drawn using online Circos plot software with the following site address: http://circos.ca/intro/tabular_visualization/$url_root/circos_online (38).

## Results

A total of 100 *E. coli* strains were isolated from broiler chickens with different conditions. The number of identified strains related to colisepticemia, YSIs, and healthy chickens was 32, 32, and 36, respectively.

### Antimicrobial Susceptibility

Antimicrobial susceptibility pattern of *E. coli* strains was determined against 15 antimicrobial agents (Supplementary Table 1). All strains were susceptible to fosfomycin, ceftriaxone, and cefixime, while 50% or more of the *E. coli* strains were resistant to other agents with the exception of gentamicin and furazolidone with resistance rates of 26 and 38%, respectively. Resistance against streptomycin, tetracycline, flumequine, and lincospectin was found in more than 80% of *E. coli* strains isolated from the diseased birds and feces of healthy chickens. In total, 98 (98%) strains were considered MDR (resistant to three or more of the tested drugs). Fecal strains exhibited a significantly greater resistance against gentamicin and enrofloxacin compared to APEC strains as represented in Supplementary Table 1 (*p* < 0.05).

### Virulence-Associated Genes

Prevalence of VAGs among *E. coli* strains is shown in Table 1. All strains (except two) harbored at least three or more VAGs. The most commonly detected genes were *tonB, csg*, and *iutA* with a prevalence of 99, 96, and 83%, respectively. Among APEC, the *iss, iutA, tsh, hly, iroN*, and *ompT* genes were significantly more likely to occur in strains from lesions of colisepticemia, whereas the *astA* gene was more prevalent among AFEC strains, as compared to YSI and CS strains (*p* < 0.05). According to Johnson et al. (36), APEC with at least four of five VAGs was considered highly virulent APEC; therefore, 91 (91%) strains can be considered potential APEC. The frequency of these profile markers was statistically significant in colisepticemic strains compared to YSI and AFEC (Table 2).

**Table 1:** Distribution of virulence-associated genes among avian *Escherichia coli* strains isolated from yolk sac infection, coliseptocemia, and fecal samples.

**Gene**	***Iss***	***iutA***	***tsh***	***papC***	***papG***	***hly***	***iroN***	***colV***	***ibe***	***csg***	***astA***	***ompT***	***tonB***
	***N* (%)**	***N* (%)**	***N* (%)**	***N* (%)**	***N* (%)**	***N* (%)**	***N* (%)**	***N* (%)**	***N* (%)**	***N* (%)**	***N* (%)**	***N* (%)**	***N* (%)**
YSI (*n* = 32)	20 (62.5)	24 (75)	14 (43.7)*	6 (18.7)	6 (18.7)	21 (65.6)	19 (59.4)	7 (21.9)	1 (3.1)	32 (100)	11 (34.4)	22 (68.7)	32 (100)
CS (*n* = 32)	29 (90.6)*	32 (100)*	25 (78.1)	4 (12.5)	4 (12.5)	31 (96.8)*	25 (78.1)*	6 (18.7)	0 (0)	32 (100)	5 (15.6)*	31 (96.8)*	32 (100)
YSI + CS (*n* = 64)	49 (76.6)	56 (87.5)	39 (60.9)	10 (15.6)	10 (15.6)	52 (81.2)	44 (68.7)	13 (20.3)	1 (1.6)	64 (100)*	16 (25)*	53 (82.8)	64 (100)
F (*n* = 36)	14 (38.9)	28 (77.8)	23 (63.9)	8 (22.2)	10 (27.8)	23 (63.9)	19 (52.8)	5 (13.9)	4 (11)	32 (88.9)*	19 (52.8)*	22 (61)	35 (97.2)
Sum (*n* = 100)	63 (63)	84 (84)	62 (62)	18 (18)	20 (20)	75 (75)	63 (63)	18 (18)	5 (5)	96 (96)	35 (35)	75 (75)	99 (99)

YSI, Yolk sac infection; CS, Colisepticemia; F, Fecal; P-values were calculated by χ^2^ test or Fisher's exact test. The values significantly higher than among the other groups are indicated as follows: ■P < 0.001, ■P < 0.01, ■P < 0.05. The values significantly lower than among the other groups (or absence) are indicated as follows: ■P < 0.001, ■P < 0.01, ■P < 0.05. For comparison among groups the highest amount of adjusted residual values if exceeding ±2 considered most significant difference observed among other cells as indicated in by (*) superscript.

**Table 2:** Frequency of predictor APEC virulence genes (*iss, iutA, hly, iroN*, and *ompT*) among yolk sac infection, colisepticemia, and fecal *Escherichia coli* strains.

**Source (No.)**	**Number of strains carry five predictor APEC genes (%)**	**Number of strains carry four or more predictor APEC genes (%)**
YSI (*n* = 32)	17 (53)	19 (59)
CS (*n* = 32)	23 (72)^*^	31 (97)^*^
YSI + CS (*n* = 64)	40 (62.5)^*^	50 (78)^*^
F (*n* = 36)	11 (30)	15 (44)

*APEC, avian pathogenic Escherichia coli; YSI, yolk sac infection; CS, colisepticemia; F, fecal*;

* *P < 0.05*.

### Phylogenetic Groups

**Figure 1:**
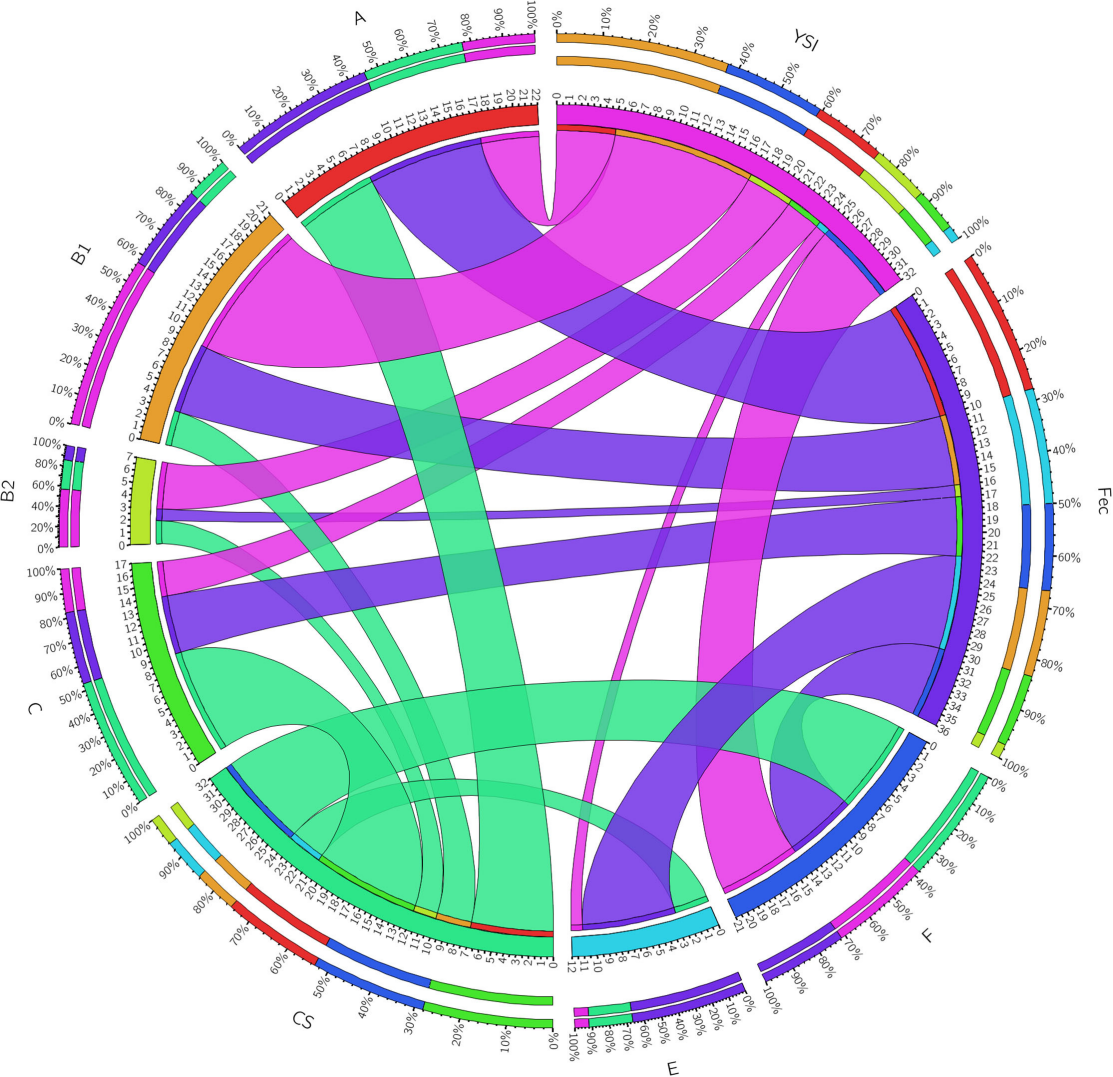
A Circos plot presenting the distribution of *Escherichia coli* strain phylogroups in YSI, yolk sak infection; CS, colisepticemia; Fec, fecal origins. The inner ring represents the number of strains in each group. The outer circle depicts distribution percentage of the traits in each group. The arc originates from strain source and terminates at the phylogenetic group. The area of each colored ribbon depicts the frequency of the strains related to the origin and phylogroup.

On phylogenetic classification, group A was the most prevalent (22%), followed by group B1 and F (21% each), and other phylotypes including C, E, and B2 with a frequency of 17, 12, and 7%, respectively (Table 3). No strains of group D and clade I were found. All phylogenetic groups were found in YSI, colisepticemic, and AFEC strains; however, the frequency of phylogroups varied according to type of *E. coli* infection group. B1 and C phylogroups were statistically significant in YSI and colisepticemic *E. coli* groups, respectively. Phylogroup A was the most frequent phylogroup in AFEC strains, whereas phylogroup E was significantly more likely to occur in AFEC strains as compared to colisepticemic and YSI strains (*p* < 0.05). Distribution of phylogroups in YSI, CS, and AFEC strains was represented as a circle using Circos plot software (Figure 1).

**Table 3:** Phylogenetic distribution of avian *Escherichia coli* strains isolated from yolk sac infection, coliseptocemia, and fecal samples.

**Phylogenetic groups**	**A (22)**	**B1 (21)**	**B2 (7)**	**C (17)**	**E (12)**	**F (21)**
YSI (*n* = 32)	5 (15.6)	12 (37.5)^*^	4 (12.5)	3 (9.4)	1 (3.1)	7 (21.9)
CS (*n* = 32)	7 (21.9)	3 (9.4)	2 (6.2)	9 (28.1)	3 (9.4)	8 (25)
YSI+CS (*n* = 64)	12 (18.8)	15 (23.4)	6 (9.4)	12 (18.8)	4 (6.2)	15 (23.4)
F (*n* = 36)	10 (27.7)	6 (16.7)	1 (2.8)	5 (13.9)	8 (22.2)^*^	6 (16.7)

*Yolk sac infection; CS, Colisepticemia; F, Fecal; P-values were calculated by χ^2^ test or Fisher's exact test. The values significantly higher than among the other groups are indicated as follows: ■P < 0.001, ■P < 0.01, ■P < 0.05. The values significantly lower than among the other groups (or absence) are indicated as follows: ■P < 0.001, ■P < 0.01, ■P < 0.05. For comparison among groups the highest adjusted residual values if exceeding +/- 2 considered most significant difference observed among other cells as indicated in by (*

* *) superscript*.

### Aggregate VAG and AMR Scores According to Phylogenetic Groups

Results showed that the group C exhibited the highest aggregate VAG score (mean: 8.7; range: 7–10) whereas the VAG scores of groups A and B1 were significantly lower than other phylogroups. Among the YSI group, the strains belonging to phylogroup B1 significantly exhibited the lowest VAG score (mean: 5.3; range: 3–9). Although phylogroup C had the highest VAG score (mean: 9), the difference, as compared to other phylogroups, was not statistically significant. Among AFEC strains, phylogroups C and A had the highest and lowest VAG scores, respectively (*p* < 0.05). Distribution of VAGs among colisepticemic *E. coli* strains belonging to different phylogroups was not statistically significant. Overall, the APEC and AFEC strains had an average of 7.2 VGs (range from 1 to 11) (Table 4).

**Table 4:** Virulence-associated genes and antimicrobial resistance scores of avian *Escherichia coli* isolates by phylogenetic groups.

	**Mean VAG scores;** ***N*** **(Mean)**					**Mean AMR scores;** ***N*** **(Mean)**				
**Phylogenetic group**	**All strains**	**YSI strains**	**CS strains**	**F strains**	***P*-value YSI + CS vs. F**	**All strains**	**YSI strains**	**CS strains**	**F strains**	***P*-value YSI + CS vs. F**
Total	100 (7.2)	32 (6.8)	32 (8)	36 (6.7)	*P* > 0.05	100 (8.4)	32 (7.25)	32 (9.1)	36 (8.8)	*P* > 0.05
A	22 (5.9)	5 (5.4)	3 (8.3)	10 (5.4)	*P* > 0.05	22 (8.7)	5 (6.8)	3 (9.7)	10 (9.3)	*P* > 0.05
B1	21 (6.2)	12 (5.3)	7 (7.6)	6 (6.3)	*P* > 0.05	21 (8.1)	12 (7.4)	7 (9.3)	6 (8.2)	*P* > 0.05
B2	7 (8)	4 (8.75)	2 (8)	1 (5)	–	7 (6.1)	4 (6.25)	2 (7)	1 (4)	–
C	17 (8.7)	3 (9)	9 (8.5)	5 (9)	*P* > 0.05	17 (9.6)	3 (8.3)	9 (10)	5 (9.6)	*P* > 0.05
E	12 (7.4)	1 (7)	3 (8)	8 (7.25)	*P* > 0.05	12 (8.7)	1 (12)	3 (9)	8 (8.1)	*P* > 0.05
F	21 (7.7)	7 (8.1)	8 (7.9)	6 (6.8)	*P* > 0.05	21 (8.3)	7 (6.7)	8 (8.4)	6 (9.7)	*P* < 0.05

*YSI, Yolk sac infection; CS, Colisepticemia; F, Fecal; VAG, Virulence-associated genes; AMR, Antimicrobial resistance; N, Number; P-values were calculated by Mann-Withny U-test. The values significantly higher than among the other groups are indicated as follows: ■P < 0.01, ■P < 0.05. The values significantly lower than among the other groups are indicated as follows: ■P < 0.001, ■P < 0.01, ■P < 0.05*.

No significant difference in the distribution of AMR scores was detected among YSI, colisepticemia, and AFEC strains belonging to different phylogenetic groups. However, among all strains, phylogroups C and B2 had the highest and lowest AMR scores, respectively (*p* < 0.05). AFEC strains in phylogroup F had higher AMR scores than the strains isolated from disease cases (YSI + colisepticemic *E. coli* strains) (*p* < 0.05). Overall, the strains had an average of 8.4 AMR score (ranging from 2 to 12) (Table 4).

### Phylogenetic Distribution of VAGs and Antimicrobial Resistance Phenotypes

Statistical analysis showed that the *iss, iutA, tsh, hly, iroN, colV*, and *ompT* genes were positively associated with group C, while *astA* gene was negatively correlated with group C (*p* < 0.05). *papC* and *papG* adhesin genes were positively associated with group B2. Furthermore, *hly* and *iroN* were also positively correlated with phylogroup B1, and *csg* and *tsh* genes were associated with groups A and E, respectively (*p* < 0.05). On the other hand, some genes were negatively correlated with phylogroups. *iss, tsh*, and *ompT* genes were negatively associated with group B1, while *iutA* was negatively correlated with group A (*p* < 0.05) (Table 5).

**Table 5:** Distribution of virulence-associated genes (VAG) and resistance to antimicrobial agents within phylogenetic groups.

**Bacterial characteristic**	**Frequency of bacterial characteristic** **within phylogenetic groups**, ***N*** **(%) of isolates**					
	**A**	**B1**	**B2**	**C**	**E**	**F**
*iss*	12 (54.5)	6 (28.6)	6 (85.7)	17 (100)^*^	5 (41.7)	17 (81)
*iutA*	13 (59.1)	16 (72.6)	7 (100)	17 (100)^*^	11 (91.7)	20 (95.2)
*tsh*	12 (54.5)	8 (38.1)	3 (49.9)	16 (94.1)^*^	11 (91.7)	12 (57.1)
*papC*	4 (18.2)	5 (23.8)	4 (57.1)^*^	1 (5.9)	2 (16.7)	2 (9.5)
*papG*	5 (22.7)	6 (28.6)	4 (57.1)^*^	1 (5.9)	2 (16.7)	2 (9.5)
*hly*	13(59.1)	12 (57.1)	6 (85.7)	17 (100)^*^	10 (83.3)	17 (81)
*iroN*	10 (45.5)	10 (42.9)	5 (71.4)	17 (100)^*^	8 (66.7)	14 (66.7)
*colV*	2 (9.1)	4 (19)	1 (14.3)	8 (47.1)^*^	1 (8.3)	2 (9.5)
*ibe*	0 (0)	0 (0)	1 (14.3)	0 (0)	1 (8.3)	3 (14.3)
*csg*	19 (86.4)^*^	20 (95.5)	7 (100)	17 (100)	12 (100)	21 (100)
*astA*	10 (45.5)	6 (28.6)	1 (14.3)	2 (11.8)^*^	7 (58.3)	9 (42.9)
*ompT*	15 (68.2)	12 (57.1)	6 (85.7)	17 (100)^*^	8 (66.7)	17 (81)
*tonB*	21 (95.5)	21 (100)	7 (100)	17 (100)	12 (100)	21 (100)
S	21 (95.5)	19 (90.5)	6 (85.7)	17 (100)	12 (100)	19 (90.5)
C	18 (81.8)	14 (66.7)	2 (28.6)^*^	14 (82.4)	8 (66.7)	12 (57.1)
N	15 (68.2)	15 (71.4)	4 (57.1)	15 (88.2)	8 (66.7)	9 (42.9)^*^
Ff	13 (59.1)	13 (61.9)	3 (42.9)	13 (76.5)	7 (58.3)	11 (52.4)
Gm	4 (18.2)	2 (9.5)	0 (0)	9 (52.9)^*^	4 (33.3)	7 (33.3)
Lp	22 (100)	19 (90.5)	6 (85.7)	16 (94.1)	12 (100)	18 (85.7)
Cz	15 (68.2)	13 (61.9)	3 (42.9)	10 (58.8)	6 (50)	15 (71.4)
Nfx	20 (90.9)	16 (76.2)	4 (57.1)	16 (94.1)	9 (75)	16 (76.2)
Sxt	18 (81.8)	18 (85.7)	4 (57.1)	15 (88.2)	9 (75)	18 (85.7)
Te	19 (86.4)	17 (81)	5 (71.4)	15 (88.2)	10 (83.3)	19 (90.5)
Fr	9 (36.4)	6 (28.6)	1 (14.3)	7 (41.2)	7 (58.3)	9 (42.9)
Fm	19 (86.4)	19 (90.5)	5 (71.4)	16 (94.1)	11 (91.7)	18 (85.7)

*Cfm, Ceftriaxone; Gm, Gentamycin; S, Streptomycin; N, Neomycin; Lp, lincospectin; Fm, Flumequine; Cr, Chloramphenicol; Fos, Fosfomycin; Nfx, Enrofloxacin; Sxt, Sulfamethoxazole-trimethoprim; Te, Tetracycline; Cro, ceftriaxone; Fr, Florfenicol; P-values were calculated by χ^2^ test or Fisher's exact test. The values significantly higher than among the other groups are indicated as follows: ■P < 0.01, ■P < 0.05. The values significantly lower than among the other groups (or absence) are indicated as follows: ■P < 0.001, ■P < 0.05. For comparison among groups the highest adjusted residual values if exceeding +/- 2 considered most significant difference observed among other cells as indicated in by (*

* *) superscript*.

Although resistance to nitrofurantoin and gentamicin was found more in strains assigned to group C and chloramphenicol and nitrofurantoin were observed less in group B2 and F, respectively, the resistance to the other antimicrobial agents had a normal distribution among phylogroups (Table 5).

### Association Between VAGs and Antimicrobial Resistance

**Figure 2:**
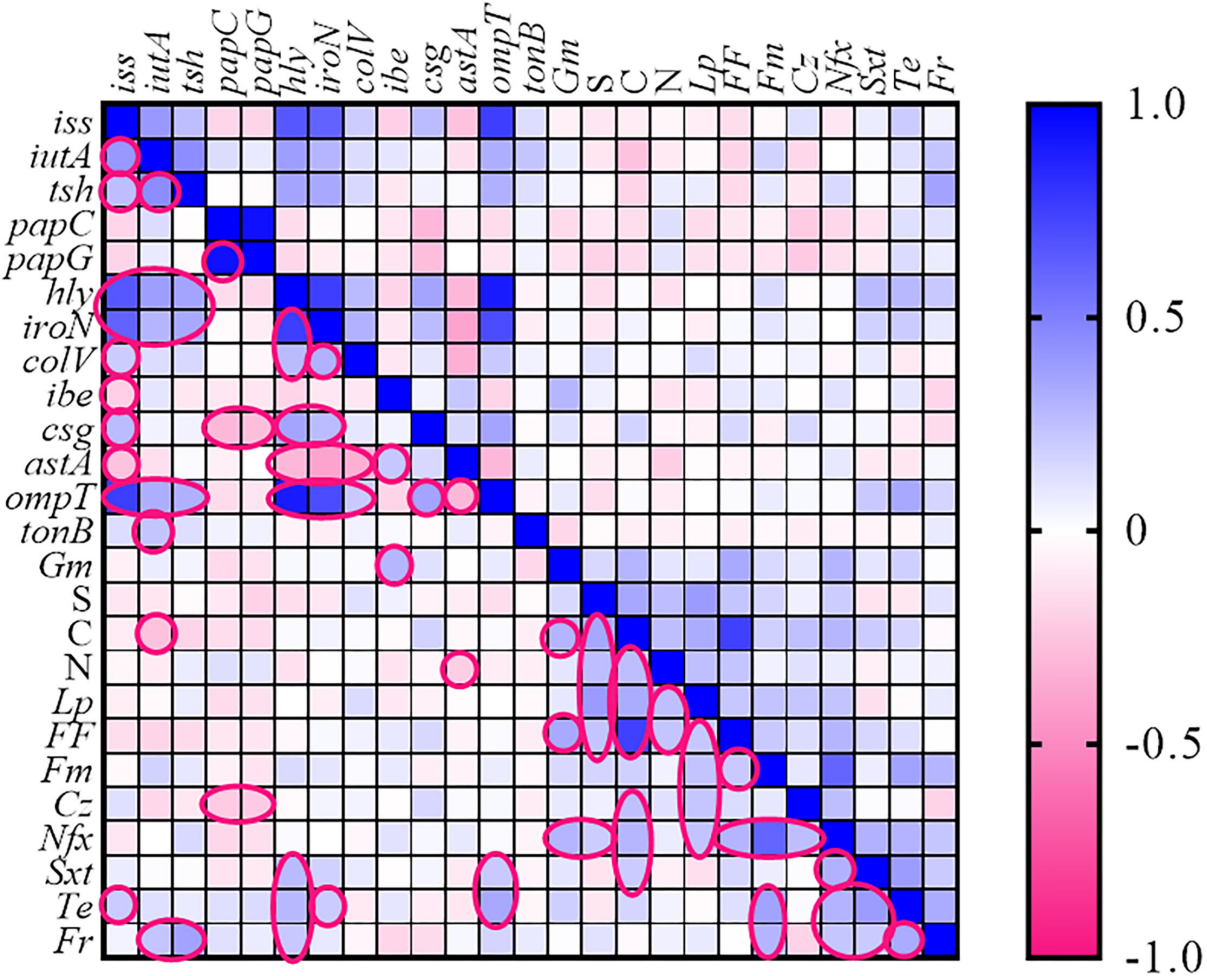
Heatmap generated according to association between genotype and phenotype traits of *Escherichia coli* strains. The statistically significant associations (*p* < 0.05) between traits are indicated in the closed pink line. No values were introduced in the cases of resistance to fosfomycin, ceftriaxone, and cefixime. Gm, gentamycin; S, streptomycin; N, neomycin; Lp, lincospectin; Fm, flumequine; Cr, chloramphenicol; Nfx, enrofloxacin; Sxt, sulfamethoxazole-trimethoprim; Te, tetracycline; Fr, florfenicol.

The association between the various VAGs and AMR phenotypes of the *E. coli* strains is shown as a heatmap (Figure 2). Spearman correlation analysis revealed a strong positive association (cutoff value was considered Spearman *R* ≥0.4) between the following genes: (*iss* with *hly, iutA, iroN*, and *ompT*), (*iutA* with *tsh*), (*papC* with *papG*), (*hly* with *iroN* and *ompT*), and (*iroN* with *ompT*) (*p* < 0.05) (for correlation coefficients, see Supplementary Table 2).

The high strong co-resistance phenomenon was observed among (chloramphenicol with florfenicol) and (enrofloxacin with flumequine) as determined in Figure 2.

### Serogrouping of *E. coli* Strains

The presence of serogroups O1, O2, O18 and O78 as the most prevalent serogroups in APEC strains was investigated. One strain was identified as O1. Five and two strains also belonged to O2 and O78, respectively. Serogroup O18 was not found among strains.

### Diversity Analysis of *E. coli* Strains Using ERIC-PCR

**Figure 3:**
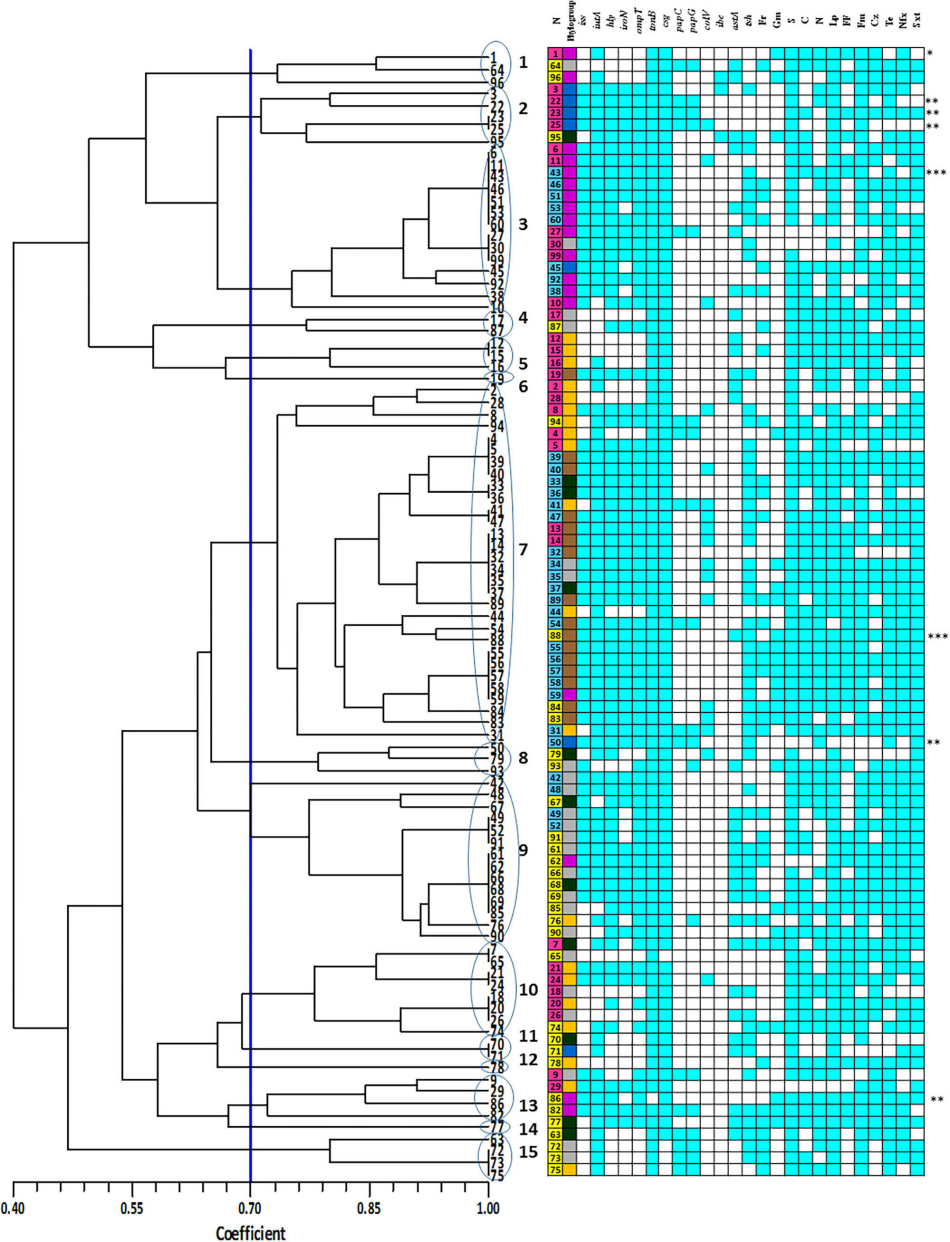
Enterobacterial repetitive intergenic consensus (ERIC) denderogram of *Escherichia coli* strains drawn using unweighted-pair group method with arithmetic mean (UPGMA). Based on a similarity index of 70% (blue line), 15 major clusters (shown by Arabic numbers) were found. Numbers at the terminal branches are strain name. The colors mean as follows: 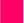: Yolk sac infection strains; 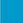: Colisepticemia strains; 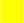: Fecal strains; 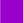: phylogroup F; 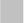: phylogroup A; 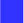: phylogroup B2; 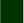: phylogroup E; 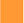: phylogroup B1; 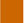: phylogroup C; Gm: gentamycin; S: streptomycin; N: neomycin; Lp: lincospectin; Fm: flumequine; Cr: chloramphenicol; Nfx: enrofloxacin; Sxt: sulfamethoxazole-trimethoprim; Te: tetracycline; Fr: florfenicol; Blue boxes indicate positive resistance phenotype or presence of virulence-associated gene. *: O1; **: O2; ***: O78.

*E. coli* strains showed various PCR bands from 230 to 2,700 bp with different patterns yielded 3 to 10 bands. Drawn dendrogram revealed 15 distinct ERIC clusters at a coefficient of 0.7 (Figure 3). Cluster 7 was the largest, having 30 (30%) *E. coli* strains aligned with 73% similarity. Approximately 94% of strains related to phylogroup C fall into this cluster, whereas there were no strains of phylogroup B2 within this cluster. More than 60% of strains in cluster 7 were isolated from colisepticemia infection. All strains of this cluster had *csg* and *tonB* genes and were resistant to streptomycin. Total drug resistance (TDR) patterns were found in two strains. Fourteen strains were placed in cluster 3. Interestingly, all were isolated from clinical specimens and 86% (12 strains) belonged to phylogroup F. The VAGs including *hly, iss, csg, ompT*, and *tonB* genes were positive in this group, whereas *ibe* was absent. Fourteen strains were also placed in cluster 9. Most strains of this cluster (71%) were AFEC and belonged to phylogroup A. The *hly, csg, ompT*, and *tonB* genes were positive in this cluster, whereas *papC, colV*, and *ibe* genes were negative. All strains also shared resistance pattern against enrofloxacin, sulfametoxazole + trimethoprim, tetracycline, and lincospectin. The rest of the clusters had <10 strains. Three (3%) strains displayed a unique profile.

### Diversity Analysis of *E. coli* Strains Using VAGs Profiles

According to the VAG profile, the strains were divided into five clusters. Cluster 3 was the largest, having 75 (75%) *E. coli* strains aligned with 70% similarity (Supplementary Figure 1). All strains that had predictor APEC genes were included in this cluster.

### Diversity of *E. coli* Strains Using Combined Genotypic and Phenotypic Traits

A dendrogram based on the presence or absence of VAGs and phenotypic resistance patterns was drawn using SIMQUAL program (Supplementary Figure 2). Based on this, the strains were divided into nine clusters. Cluster B was the largest with 68 strains (68%) aligned with 76% similarity. Approximately 93, 93, and 87% of *E. coli* strains that belonged to clusters 3, 9, and 7 of ERIC-PCR fall into the cluster B, respectively. All phylogroup C *E. coli* strains were placed in this cluster.

## Discussion

Colibacillosis is an important disease in the poultry industry caused by *E. coli*. However, unlike most enteric pathogenic *E. coli*, the extraintestinal infections are not specifically attributed to few well-known virulence-associated determinants. In the present study, we aimed to reach a more integrated perspective on APEC strains by considering various genotypic and phenotypic traits and performing statistical analysis to search for any possible associations. Any observed association could be considered a primary clue that could be further evaluated in future studies. For this purpose, we characterized and compared the strains isolated from sick and healthy birds according to AMR phenotype, VAGs, phylogenetic group, and ERIC fingerprints. All investigated *E. coli* strains were susceptible to fosfomycin, ceftriaxone, and cefixime; however, the rate of resistance against other antimicrobial agents was alarming. Despite the prohibition of some antibiotics in veterinary medicine such as chloramphenicol, 65% of the investigated strains were resistant to this drug. In the latest edition of World Health Organization Critically Important Antimicrobials lists (WHO CIA lists; 6th version), the highest priority CIAs are follows: third- and higher-generation cephalosporins, polymyxins, quinolones, macrolides-ketolides, and glycopeptides (39). Ceftriaxone and cefixime are the third generation of cephalosporins and no resistance was found to them. However, more than 80% of *E. coli* strains tested were resistant to fluoroquinolones (enrofloxacin and flumequine), which seems to be alarming. The use of antimicrobial agents as growth-promoting additives in poultry is not recommended by the Iranian Veterinary Organization, but we have no official information about illegal antibiotic usage as growth promoter in the poultry industry. However, Shahroozian and Khoshgoftar (40) found gentamicin residue in 78.57% of poultry meat samples of Semnan province (40). In another study, enrofloxacin residue was detected in poultry meat (41). Such antibiotic residues may be due to the unofficial use of drugs with the unfortunate outcome of selection of resistance among intestinal commensal *E. coli* strains. Our results about high AMR and MDR rates in AFEC and APEC strains were consistent with previous studies in Iran (42, 43). Importantly, the resistance rates were significantly higher in fecal commensal strains (AFEC) against some antimicrobials such as gentamycin and enrofloxacin; this observation indicates the necessity of surveillance of AMR in animals in both commensal and pathogenic strains particularly where antimicrobial stewardship strategies have not been rigorously implemented.

In the present study, we investigated the frequency and scores of VAGs among different *E. coli* strains and the association between these genes. *E. coli* strains isolated from chickens with typical signs of colibacillosis were divided into two groups of colisepticemic and YSI. Statistical analysis revealed that colisepticemic strains significantly have more APEC predictors genes (*iss, iutA, hly, iroN*, and *ompT*) introduced by Johnson et al. (36) compared to YSI strains (Table 2). In addition, the *tsh* gene was also attributed to the colisepticemic strains. However, more than 63% of AFEC strains also harbored the *tsh* gene. This gene is known as a putative virulence gene (44), which is located on the variable region of ColV plasmids, non-ColV plasmids, or within PAIs on the bacterial chromosome such as PAI III_536_ that was originally identified in UPEC strains (14). Compression of the frequency of VAGs and VAG scores show that YSI-causing strains have less virulence traits than colisepticemic strains; therefore, the disease caused by these strains could be secondary in nature. The *astA* gene was more prevalent in the AFEC strains compared to APEC. Although *astA* has been reported in ExPEC (45), it appears to be more related to intestinal (commensal or diarrheagenic) strains (46–48). *astA* is a virulence gene also associated with the enteroaggregative *E. coli* (EAEC) isolates (49–51), although it is not restricted to this pathotype (52). The presence of this gene does not appear to contribute to disease by *E. coli* unless combined with other virulence traits (53).

The frequency of *colV* gene was not statistically significant between two groups of APEC and AFEC strains, and this gene was not detected in 69% of strains with predictor VAGs of APEC. Although the *colV* operon is the namesake trait of ColV plasmids and encodes the production of colicins, it is not necessarily related to APEC pathogenicity. The absence of *colV* gene in some APEC strains with all predictor genes indicates that these markers may not be solely carried on the ColV plasmids. Johnson et al. (13) found that the ColBM plasmids can be the location of VAGs occurring in ColV-negative strains. Therefore, the absence of *colV* gene does not indicate that the strains are not pathogenic.

Horizontal gene transfer or carriage within the mobile genetic elements, or co-selection of virulence genes leads to the simultaneous appearance of these genes in a particular strain (32, 54, 55). Since several virulence factors are usually involved in causing infection and disease, understanding these relationships is very important to unraveling the pathogenesis of colibacillosis. Correlation analysis revealed the associations among *iss, hly, iutA, iroN*, and *ompT* VAGs. This gene profile appears to play an important role in the pathogenicity of APEC, as previously identified in research by Johnson et al. (14). We also found a positive association between *tsh* and other APEC predictor genes; however, the correlation between *tsh* with *iutA* was stronger than the other genes. *iutA* encodes the ferric aerobactin receptor, which is involved in acquisition of iron. A group of strains that were positive for both *iutA*/*tsh* and negative for other predictor genes were placed in the AFEC group. Therefore, the association of these two genes seems insufficient to predict the pathogenic potential of APEC. Very strong association was found between *papG* and *papC* genes, which are part of the *pap* operon within a PAI on the chromosome (56).

Regarding the relationship between AMR and VAGs, two possibilities have been raised. First, resistant strains are more virulent because many genetic elements such as plasmids, integrons, and composite transposons may carry both VAGs and AMR genes simultaneously. On the other hand, some researchers believe that AMR strains are less virulent than susceptible ones because AMR and virulence have not necessarily evolved simultaneously (23, 57). In the present study, no relationship was found between VAGs and resistance phenotype. Only a weak positive association was found between *ompT* and resistance to tetracycline and *tsh* and furazolidone. The particular host, geographical origin, and type of antimicrobial agent used, can probably affect the relationship between AMR and VAGs (58).

A strong cross-resistance phenomenon was observed between chloramphenicol with florfenicol. Florfenicol is an analog of chloramphenicol that has not been approved for human use. It was reported that cross-resistance to chloramphenicol and florfenicol is due to non-enzymatic activity of *E. coli flo* gene. Also, a strong cross-resistance was found between enrofloxacin (quinolone) and flumequine (fluoroquinolone). Resistance against quinolones and fluoroquinolones is acquired through mutations in some chromosomal genes such as *gyrA* and *parC* or by acquiring a wide range of plasmid-encoded genes (59).

Classification of strains based on phylogenetic group showed that AFEC strains mostly belonged to groups A and E, while colisepticemic and YSI strains belonged to groups C and B1, respectively. Phylogroup A had the lowest VS among other phylogroups of AFEC strains. In contrast, while only three colisepticemic strains were present in the A group, their VS mean (8.3) was high, which indicates inconsistency of VSs within a particular phylogroup. The highest mean of VS was found in group C and most strains of this phylogroup were isolated from colisepticemic lesions. Although few reports indicated that this phylogroup is close to groups B1 or A (60, 61), the present study showed that the strains related to phylogroup C are virulent and play an important role in development of colibacillosis. Instead, phylogroup B1 is more common in commensal strains and has less virulence. The B1 group strains isolated from YSI lesions had the lowest VS and had a negative association with two main APEC virulence genes (*iss* and *ompT*). Within the B2 phylogroup, the *papC* and *papG* genes were significantly more prevalent. *pap* gene clusters are located on the bacterial chromosome and are common virulence genes of group B2 UPEC strains. It should be noted that recent studies comparing the genomic features of APEC showed the multilineage evolution of pathogenic strains that makes comparison difficult. For example, O78 belongs to two lineages in phylogroups C and G (ST-23 and ST-117) and O1, and O2 belongs to three subpopulations within the third lineage in the B2 phylogroup (ST-95, ST-140, and ST-428/429) (62). Several studies have found that the APEC and UPEC strains are phylogenetically close and share some of the same VAGs and therefore should be considered potential zoonoses (7, 62–64).

Cluster analysis by UPGMA was performed on obtained data of ERIC-PCR, VAG-AMR traits, and VAGs to determine which assay is more suitable for the classification of strains. By ERIC-PCR, 15 clusters were identified. An integrated analysis revealed that, in many cases, the strains belonging to one cluster have high similarity in terms of virulence, resistance, source, and phylogenetic group. Cluster 7 contained the majority of phylogenetic group C that were isolated from colisepticemic lesions and also had high scores of virulence and AMR. Cluster 9 was mostly composed of AFEC strains and belonged to group A, with relatively high virulence and resistance scores. The strains that belonged to clusters 2 and 5 shared a similar origin and phylogenetic group and almost similar VAGs. Therefore, ERIC-PCR could be a practical approach for the initial classification of the strains in epidemiological studies. However, there are different viewpoints on ERIC-PCR discriminatory power for clonal differentiation. Some studies suggested the ERIC-PCR for investigation of clonal diversity and association between the phenotype and genotype of APEC strains (65, 66), while other researchers disagree (67).

## Conclusion

In the present study, we tried to have a bird's eye view on the complicated APEC pathotype. Our finding indicates that virulence and AMR are not essentially related traits in avian *E. coli* and commensal strains may show greater resistance in some instances. This shows that resistance and virulence could evolve divergently, and therefore, AMR monitoring of poultry farms cannot only rely on the recovered strains of diseased birds. This is of particular interest where antimicrobial stewardship strategies have not been rigorously implemented. In the present study, strains related to phylogenetic group C had higher virulence and AMR scores compared to other groups, which indicates the importance of this phylogroup in both disease burden and AMR problem. Shared VAG reservoirs of APEC/AFEC and UPEC strains propose that avian *E. coli* could be potential reservoirs of VAGs for ExPEC strains in humans and therefore is a potential zoonotic agent. Further integrated studies on a larger number of strains along with assessment of more detailed genotypic features could potentially help us to upgrade our knowledge on virulence, resistance, and evolution of ExPEC.

## Data Availability Statement

The original contributions presented in the study are included in the article/Supplementary Material, further inquiries can be directed to the corresponding author/s.

## Ethics Statement

Ethical review and approval was not required for the animal study because Since we did not work on animals and the isolates were available from previously approved study re-submission for new approval was not necessary.

## Author Contributions

SR and MA designed the study and analyzed the data. MA, AN, and MS performed the study. SR wrote the first draft. SR, MA, and SP contributed to writing and critically reviewed the manuscript and assisted in analysis of data. All authors contributed to the article and approved the submitted version.

## Conflict of Interest

The authors declare that the research was conducted in the absence of any commercial or financial relationships that could be construed as a potential conflict of interest.

## Publisher's Note

All claims expressed in this article are solely those of the authors and do not necessarily represent those of their affiliated organizations, or those of the publisher, the editors and the reviewers. Any product that may be evaluated in this article, or claim that may be made by its manufacturer, is not guaranteed or endorsed by the publisher.

## Supplementary Material

Supplementary Figure 1 Unweighted pair-group method with arithmetic clustering (UPGMA) dendrogram based on data from virulence-associated gene profiles of *Escherichia coli* strains. Based on a similarity index of 63% (blue line), 5 majors clusters (I-V) were found. The colors mean as follow: : Yolk sac infection strains; : Clisepticemia strains; : Fecal strains; : phlogroup F; : phlogroup A; : phlogroup B2; : phlogroup E; : phylogroup B1; : phlogroup C; ERIC: Enterobacterial repetitive intergenic consensus. Blue boxes indicate presence of virulence-associated gene. *: O1; **: O2; ***: O78.

Supplementary Figure 2 Unweighted pair-group method with arithmetic clustering (UPGMA) dendrogram based on data from antimicrobial resistance phenotype and virulence-associated gene profiles of *Escherichia coli* strains. Based on a similarity index of 70% (black line), 9 majors clusters (A-I) were found. The colors mean as follow: : Yolk sac infection strains; : Clisepticemia strains; : Fecal strains; : phlogroup F; : phlogroup A; : phlogroup B2; : phlogroup E; : phylogroup B1; : phlogroup C; ERIC: Enterobacterial repetitive intergenic consensus. *: O1; **: O2; ***: O78.

Supplementary Table 1 Antimicrobial resistance among avian *Escherichia coli* strains isolated from samples of diseased birds and feces of healthy chickens.

Supplementary Table 2 The *P* values of associations between different traits of avian *Escherichia coli* strains.
