# Supplementary figures and images for: An Integrated Perspective on Virulence-Associated Genes (VAGs), Antimicrobial Resistance (AMR), and Phylogenetic Clusters of Pathogenic and Non-pathogenic Avian Escherichia coli
Source: Front Vet Sci. 2021 Nov 24;8:758124. doi: 10.3389/fvets.2021.758124 (PMC8651559; doi:10.3389/fvets.2021.758124)

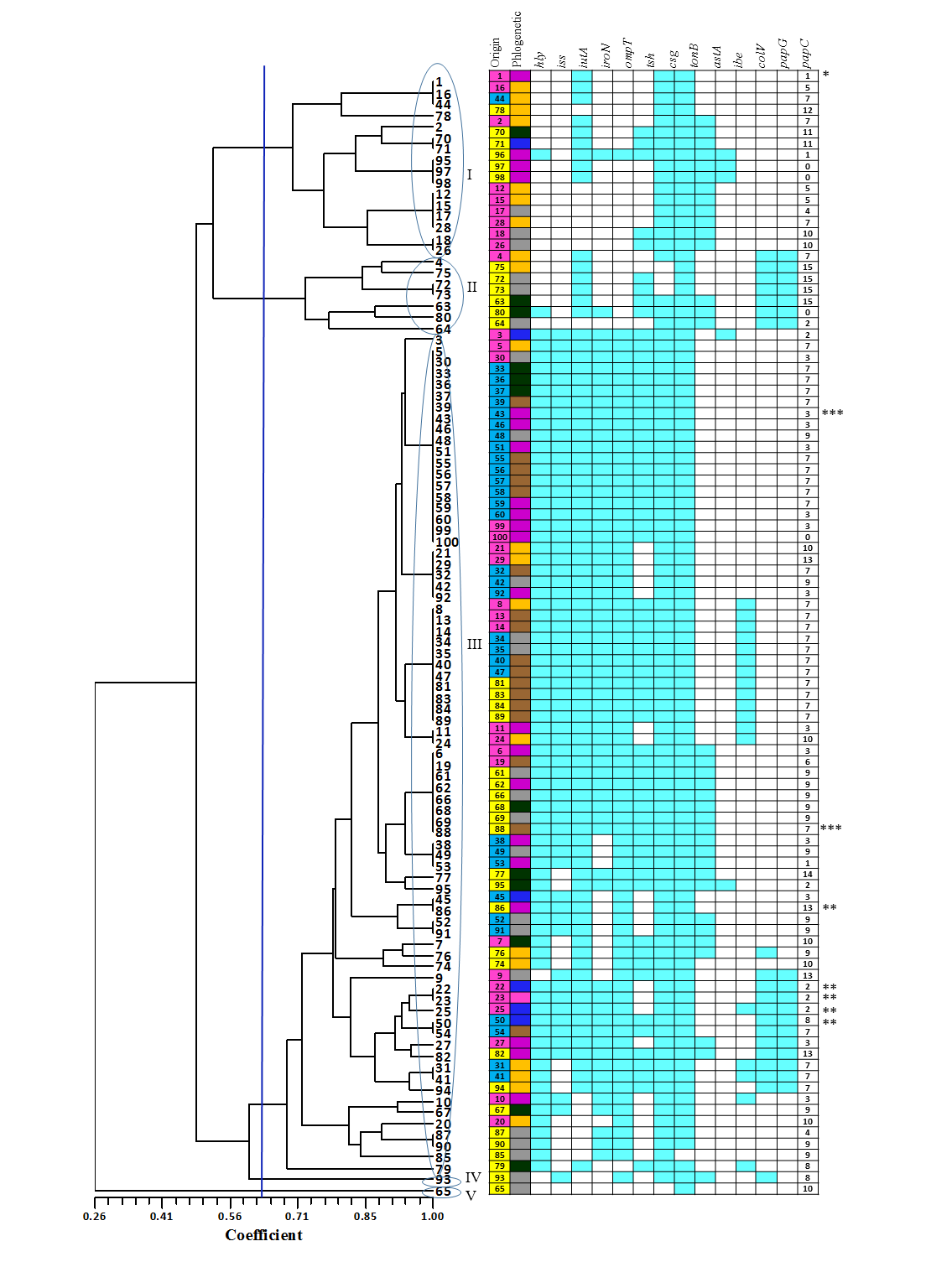

Supplement: Supplementary Figure 1 — Unweighted pair-group method with arithmetic clustering (UPGMA) dendrogram based on data from virulence-associated gene profiles of Escherichia coli strains. Based on a similarity index of 63% (blue line), 5 majors clusters (I-V) were found. The colors mean as follow: : Yolk sac infection strains; : Clisepticemia strains; : Fecal strains; : phlogroup F; : phlogroup A; : phlogroup B2; : phlogroup E; : phylogroup B1; : phlogroup C; ERIC: Enterobacterial repetitive intergenic consensus. Blue boxes indicate presence of virulence-associated gene. *: O1; **: O2; ***: O78. [file Image_1.TIF]

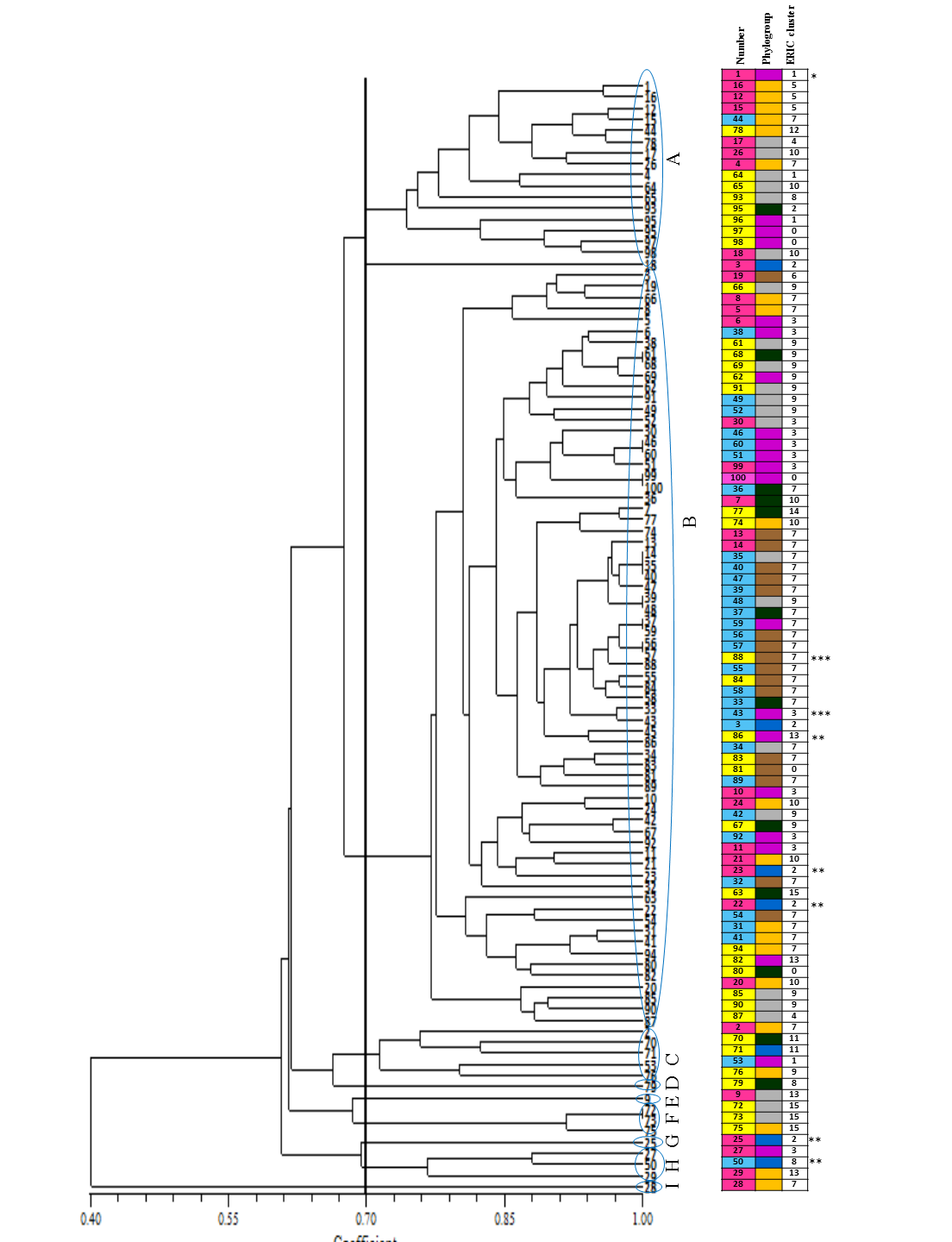

Supplement: Supplementary Figure 2 — Unweighted pair-group method with arithmetic clustering (UPGMA) dendrogram based on data from antimicrobial resistance phenotype and virulence-associated gene profiles of Escherichia coli strains. Based on a similarity index of 70% (black line), 9 majors clusters (A-I) were found. The colors mean as follow: : Yolk sac infection strains; : Clisepticemia strains; : Fecal strains; : phlogroup F; : phlogroup A; : phlogroup B2; : phlogroup E; : phylogroup B1; : phlogroup C; ERIC: Enterobacterial repetitive intergenic consensus. *: O1; **: O2; ***: O78. [file Image_2.TIF]
