# Supplementary material for: An Integrated Perspective on Virulence-Associated Genes (VAGs), Antimicrobial Resistance (AMR), and Phylogenetic Clusters of Pathogenic and Non-pathogenic Avian Escherichia coli
Source: Front Vet Sci. 2021 Nov 24;8:758124. doi: 10.3389/fvets.2021.758124 (PMC8651559; doi:10.3389/fvets.2021.758124)
Supplement: Supplementary Table 1 — Antimicrobial resistance among avian Escherichia coli strains isolated from samples of diseased birds and feces of healthy chickens. [file Table_1.docx]

**Supplementary Table 1.** Antimicrobial resistance among avian *Escherichia coli* strains isolated from samples of diseased birds and feces of healthy chickens

| **Antimicrobial agents** | **Cfm N (%)** | **Gm N (%)** | **S N (%)** | **C N (%)** | **N N (%)** | **Lp N (%)** | **Ff N (%)** | **Fm N (%)** | **Cz N (%)** | **Fos N (%)** | **Nfx N (%)** | **Sxt N (%)** | **Te N (%)** | **Cro N (%)** | **Fr N (%)** |
| --- | --- | --- | --- | --- | --- | --- | --- | --- | --- | --- | --- | --- | --- | --- | --- |
| **YSI+CS (n=64)** | 0 (0) | 10 (15.6) | 60 (93.7) | 41 (64) | 45 (69.2) | 57 (89) | 39 (60.9) | 55 (86) | 43 (67.2) | 0 (0) | 48 (75) | 51 (79.7) | 52 (81.2) | 0 (0) | 21 (32.8) |
| **F (n=36)** | 0 (0) | 16 (44.4) | 34 (94.4) | 24 (66.6) | 20 (55.5) | 35 (97.2) | 22 (61.1) | 33 (91.7) | 19 (52.7) | 0 (0) | 33 (91.6) | 31 (86.1) | 33 (91.6) | 0 (0) | 17 (47.2) |
| **Total (n=100)** | 0 (0) | 26 (26) | 94 (94) | 65 (65) | 65 (65) | 92 (92) | 61 (61) | 88 (88) | 62 (62) | 0 (0) | 81 (81) | 82 (82) | 85 (85) | 0 (0) | 38 (38) |

YSI: Yolk sac infection; CS: Colisepticemia; F: Fecal; Cfm: Ceftriaxone; Gm: Gentamycin; S: Streptomycin; N: Neomycin; Lp: lincospectin; Fm: Flumequine; Cr: Chloramphenicol; Fos: Fosfomycin; Nfx: Enrofloxacin; Sxt: Sulfamethoxazole-trimethoprim; Te: Tetracycline; Cro: ceftriaxone; Fr: Florfenicol; *P* values were calculated by χ2 test or Fisher’s exact test. The values significantly higher than among the other groups are indicated as follows: ◼P < 0.01, ◼P < 0.05. The values significantly lower than among the other groups are indicated as follows: ◼P < 0.01, ◼P < 0.05.
