# Supplementary material for: An Integrated Perspective on Virulence-Associated Genes (VAGs), Antimicrobial Resistance (AMR), and Phylogenetic Clusters of Pathogenic and Non-pathogenic Avian Escherichia coli
Source: Front Vet Sci. 2021 Nov 24;8:758124. doi: 10.3389/fvets.2021.758124 (PMC8651559; doi:10.3389/fvets.2021.758124)
Supplement: Supplementary Table 2 — The P values of associations between different traits of avian Escherichia coli strains. [file Table_2.docx]

|  | *iss* | *iutA* | *tsh* | *papC* | *papG* | *hly* | *iroN* | *colV* | *ibe* | *csg* | *astA* | *ompT* | *tonB* | Gm | S | C | N | Lp | Ff | Fm | Cz | Nfx | Sxt | Te | Fr |
| --- | --- | --- | --- | --- | --- | --- | --- | --- | --- | --- | --- | --- | --- | --- | --- | --- | --- | --- | --- | --- | --- | --- | --- | --- | --- |
| *iss* | 1 | 0.40 | 0.25 | -0.18 | -0.18 | 0.65 | 0.61 | 0.19 | -0.2 | 0.26 | -0.26 | 0.75 | 0.13 | -0.06 | -0.1 | -0.08 | -0.04 | -0.07 | -0.14 | -0.02 | 0.12 | -0.1 | 0.07 | 0.2 | 0.04 |
| *iutA* | 0.4 | 1 | 0.44 | 0.13 | 0.08 | 0.37 | 0.28 | 0.13 | 0.1 | 0.05 | -0.13 | 0.31 | 0.23 | 0.07 | -0.11 | -0.26 | -0.09 | -0.02 | -0.18 | 0.17 | -0.179 | -0.002 | 0.008 | 0.12 | 0.22 |
| *tsh* | 0.25 | 0.44 | 1 | -0.008 | -0.02 | 0.35 | 0.33 | 0.15 | -0.10 | 0.05 | 0.01 | 0.30 | 0.12 | 0.04 | -0.02 | -0.18 | 0.07 | 0.07 | -0.16 | 0.09 | -0.107 | 0.14 | 0.008 | 0.07 | 0.35 |
| *papC* | -0.18 | 0.13 | -0.008 | 1 | 0.93 | -0.15 | -0.01 | -0.01 | -0.10 | -0.30 | -0.07 | -0.15 | 0.04 | -0.15 | -0.10 | -0.14 | 0.12 | -0.14 | -0.10 | -0.06 | -0.22 | -0.17 | -0.11 | 0.12 | 0.11 |
| *papG* | -0.18 | 0.08 | -0.02 | 0.93 | 1 | -0.17 | -0.08 | -0.03 | -0.11 | -0.28 | 0 | -0.11 | 0.05 | -0.12 | -0.18 | -0.15 | 0.10 | -0.12 | -0.11 | -0.12 | -0.22 | -0.14 | -0.09 | 0.14 | 0.07 |
| *hly* | 0.65 | 0.37 | 0.35 | -0.15 | -0.17 | 1 | 0.75 | 0.27 | -0.18 | 0.35 | -0.30 | 0.89 | -0.05 | 0.02 | -0.14 | 0.01 | -0.13 | 0 | -0.03 | 0.14 | -0.02 | 0.01 | 0.27 | 0.27 | 0.21 |
| *iroN* | 0.61 | 0.28 | 0.33 | -0.01 | -0.08 | 0.75 | 1 | 0.30 | -0.10 | 0.26 | -0.39 | 0.70 | -0.07 | 0.02 | -0.10 | 0.04 | 0.002 | -0.07 | 0.02 | 0.09 | 0.04 | -0.001 | 0.18 | 0.20 | 0.08 |
| *colV* | 0.19 | 0.13 | 0.15 | -0.01 | -0.03 | 0.27 | 0.30 | 1 | -0.10 | 0.09 | -0.34 | 0.21 | 0.04 | 0.01 | 0.11 | 0.01 | 0.01 | 0.13 | 0.05 | 0.01 | 0.04 | -0.03 | 0.08 | -0.09 | -0.04 |
| *ibe* | -0.2 | 0.10 | -0.10 | -0.10 | -0.11 | -0.18 | -0.10 | -0.10 | 1 | 0.04 | 0.21 | -0.18 | 0.02 | 0.28 | 0.05 | -0.02 | -0.12 | -0.10 | 0.08 | 0.08 | -0.009 | 0.11 | -0.01 | 0.09 | -0.17 |
| *csg* | 0.26 | 0.05 | 0.05 | -0.30 | -0.28 | 0.35 | 0.26 | 0.09 | 0.04 | 1 | 0.14 | 0.35 | -0.02 | 0.12 | -0.05 | 0.17 | -0.04 | -0.06 | 0.15 | -0.07 | 0.15 | 0.03 | 0.03 | -0.08 | -0.15 |
| *astA* | -0.26 | -0.13 | 0.01 | -0.07 | 0 | -0.30 | -0.39 | -0.34 | 0.21 | 0.14 | 1 | -0.30 | 0.07 | -0.04 | -0.07 | -0.03 | -0.20 | -0.01 | -0.05 | -0.05 | 0.012 | 0.08 | -0.09 | -0.10 | 0.03 |
| *ompT* | 0.75 | 0.31 | 0.30 | -0.15 | -0.11 | 0.89 | 0.70 | 0.21 | -0.18 | 0.35 | -0.3 | 1 | -0.05 | 0.07 | -0.14 | 0.01 | -0.08 | 0 | 0.01 | 0.07 | 0.02 | 0.01 | 0.21 | 0.33 | 0.16 |
| *tonB* | 0.13 | 0.23 | 0.12 | 0.04 | 0.05 | -0.05 | -0.07 | 0.04 | 0.02 | -0.02 | 0.07 | -0.05 | 1 | -0.16 | -0.02 | -0.07 | -0.07 | -0.02 | -0.08 | -0.03 | -0.07 | -0.04 | -0.04 | -0.04 | 0.07 |
| Gm | -0.06 | 0.07 | 0.04 | -0.15 | -0.12 | 0.02 | 0.02 | 0.01 | 0.28 | 0.12 | -0.04 | 0.07 | -0.16 | 1 | 0.14 | 0.29 | 0.10 | 0.09 | 0.33 | 0.14 | 0.08 | 0.28 | 0.09 | 0.18 | 0.005 |
| S | -0.10 | -0.11 | -0.024 | -0.10 | -0.18 | -0.14 | -0.10 | 0.11 | 0.05 | -0.05 | -0.07 | -0.14 | -0.02 | 0.14 | 1 | 0.34 | 0.25 | 0.39 | 0.22 | 0.16 | 0.06 | 0.19 | -0.11 | -0.10 | 0.11 |
| C | -0.08 | -0.26 | -0.18 | -0.14 | -0.15 | 0.01 | 0.04 | 0.01 | -0.02 | 0.17 | -0.03 | 0.01 | -0.07 | 0.29 | 0.34 | 1 | 0.25 | 0.32 | 0.74 | 0.18 | 0.24 | 0.28 | 0.20 | 0.16 | -0.03 |
| N | -0.04 | -0.09 | 0.07 | 0.12 | 0.10 | -0.13 | 0.002 | 0.01 | -0.12 | -0.04 | -0.20 | -0.08 | -0.07 | 0.10 | 0.25 | 0.25 | 1 | 0.24 | 0.22 | 0.05 | 0.11 | 0.07 | -0.07 | 0.04 | 0.05 |
| Lp | -0.07 | -0.02 | 0.07 | -0.14 | -0.12 | 0 | -0.07 | 0.13 | -0.10 | -0.06 | -0.01 | 0 | -0.02 | 0.09 | 0.39 | 0.32 | 0.24 | 1 | 0.21 | 0.23 | 0.22 | 0.23 | -0.13 | -0.02 | 0.07 |
| FF | -0.14 | -0.18 | -0.16 | -0.10 | -0.11 | -0.03 | 0.02 | 0.05 | 0.08 | 0.15 | -0.05 | 0.01 | -0.08 | 0.33 | 0.22 | 0.74 | 0.22 | 0.21 | 1 | 0.20 | 0.13 | 0.29 | 0.15 | 0.12 | -0.007 |
| Fm | -0.02 | 0.17 | 0.09 | -0.06 | -0.12 | 0.14 | 0.09 | 0.01 | 0.08 | -0.07 | -0.05 | 0.07 | -0.03 | 0.14 | 0.16 | 0.18 | 0.05 | 0.23 | 0.20 | 1 | 0.091 | 0.60 | 0.06 | 0.36 | 0.28 |
| Cz | 0.12 | -0.17 | -0.10 | -0.22 | -0.22 | -0.02 | 0.04 | 0.04 | -0.09 | 0.15 | 0.01 | 0.02 | -0.07 | 0.08 | 0.06 | 0.24 | 0.11 | 0.22 | 0.13 | 0.09 | 1 | 0.25 | 0.008 | 0.017 | -0.19 |
| Nfx | -0.10 | -0.002 | 0.14 | -0.17 | -0.14 | 0.01 | -0.001 | -0.03 | 0.11 | 0.03 | 0.08 | 0.01 | -0.04 | 0.28 | 0.19 | 0.28 | 0.072 | 0.23 | 0.29 | 0.60 | 0.25 | 1 | 0.303 | 0.29 | 0.22 |
| Sxt | 0.07 | 0.008 | 0.008 | -0.11 | -0.09 | 0.27 | 0.18 | 0.08 | -0.01 | 0.03 | -0.09 | 0.21 | -0.04 | 0.09 | -0.11 | 0.20 | -0.07 | -0.13 | 0.15 | 0.06 | 0.008 | 0.30 | 1 | 0.38 | 0.20 |
| Te | 0.20 | 0.12 | 0.07 | 0.12 | 0.14 | 0.27 | 0.20 | -0.09 | 0.09 | -0.08 | -0.10 | 0.33 | -0.04 | 0.18 | -0.10 | 0.16 | 0.04 | -0.02 | 0.12 | 0.36 | 0.017 | 0.29 | 0.38 | 1 | 0.32 |
| Fr | 0.04 | 0.22 | 0.35 | 0.11 | 0.07 | 0.21 | 0.08 | -0.04 | -0.17 | -0.15 | 0.03 | 0.16 | 0.07 | 0.005 | 0.11 | -0.03 | 0.05 | 0.07 | -0.007 | 0.28 | -0.19 | 0.22 | 0.20 | 0.32 | 1 |

Supplementary Table 2. The *P* values of associations between different traits of avian *Escherichia coli* strains
